# Safflor yellow B reduces hypoxia-mediated vasoconstriction by regulating endothelial micro ribonucleic acid/nitric oxide synthase signaling

**DOI:** 10.18632/oncotarget.20133

**Published:** 2017-08-10

**Authors:** Chaoyun Wang, Ying Yang, Miao Li, Xin Liu, Qiaoyun Wang, Wenyu Xin, Hongliu Sun, Qingyin Zheng

**Affiliations:** ^1^ School of Enology, Binzhou Medical University, Yantai 264003, P.R. China; ^2^ School of Pharmacy, Binzhou Medical University, Yantai 264003, P.R. China; ^3^ Department of Otolaryngology-Head and Neck Surgery, Case Western Reserve University, Cleveland, OH 44106, USA

**Keywords:** HIF-1α, hypoxia, miR-199a, miR-138, NO synthase

## Abstract

Hypoxia-induced generation of vasoconstrictors reduces cerebral blood flow (CBF) while nitric oxide (NO) synthase (NOS) and microRNAs (miRNA) in endothelial cells (ECs) suppress vasoconstriction. Safflor yellow B (SYB), a natural plant compound, previously attenuated angiotensin II-mediated injury of ECs and maintained endothelial function. This study investigated the putative involvement of NOS and miRNAs in SYB-mediated resistance to hypoxia-induced vasoconstriction. *In vivo*, chronic hypoxia was induced in rats, and SYB was administered intravenously. *In vitro*, rat primary aortic ECs were cultured under oxygen and glucose deprivation. After treatment with anti-microR-199a, as well as the NOS inhibitor, N(G)-nitro-L-arginine methyl ester, SYB, or both, cell viability, NO and peroxynitrite (ONOO-) levels, NOS expression, and miRNA levels were evaluated. SYB significantly alleviated hypoxia-mediated vasoconstriction and increased CBF endothelium-dependently. SYB upregulated miR-199a, increased EC viability, decreased endothelin-1 (ET-1) levels, inhibited protein kinase C (PKC) activity, and suppressed hypoxia inducible factor-1α (HIF-1α) expression. Furthermore, the SYB-mediated reduction of inducible NOS reduced ONOO- levels. In addition, SYB downregulated miR-138 and, thereby, enhanced S100A1 and endothelial NOS activity. Hypoxia-mediated regulation of miR-138 and miR-199a inhibited endothelial NOS expression and activation, which triggered ET-1 release and vasoconstriction. Therefore, SYB treatment reduced hypoxia-induced vasoconstriction through miR-199a/endothelial NOS signaling.

## INTRODUCTION

The nitric oxide (NO) generated and released by endothelial NO synthase (eNOS) in endothelial cells (ECs) exerts multiple beneficial effects on vessels and plays a critical role in maintaining cardiovascular homeostasis [1]. NO is a scavenger that effectively counteracts hypoxia-mediated increases in reactive oxygen species (ROS) levels [2, 3]. The appropriate expression and activity of eNOS are critically important in blood vessel wall function and represent a sensitive and highly effective system for maintaining local blood flow to organs [4, 5]. Previous studies have shown that hypoxia can cause endothelial dysfunction, thereby promoting vasoconstriction and thrombosis, which reduces blood flow [6, 7]. Furthermore, these effects occur via downregulation of *eNOS* gene expression and inhibition of NO generation in ECs [6, 7].

Endothelin-1 (ET-1) is involved in vasoconstriction and the regulation of blood pressure [8]. Hypoxia/ischemia aggravates the vascular response to ET-1, which increases cerebral vasoconstriction and decreases the cerebral blood flow (CBF) [9, 10]. Hypoxia upregulates *ET-1* gene expression in ECs by activating hypoxia inducible factor-1α (HIF-1α) [11, 12]. In addition, NO was shown to enhance the HIF-1α stabilization induced by hypoxia [13]. However, it is not clear whether eNOS influences ET-1 expression by regulating NO production during hypoxia. Hypoxia significantly downregulates *eNOS* gene expression in ECs, and the post-transcriptional downregulation of eNOS mRNA levels contributes considerably to this effect [14]. microRNAs (miRNAs) are small (∼ 22 nucleotides) phylogenetically conserved noncoding single-stranded RNA molecules that bind to target mRNAs and function as potent post-transcriptional regulators of mRNA stability and translation [15–17]. miR-138 downregulates eNOS activity by acting on the Ca^2+^ binding protein S100A1 [18]. miR-199a affects HIF-1α translation [19] and, thereby, inhibits iNOS activity and ET-1 generation [19, 20].

**Figure 1:**
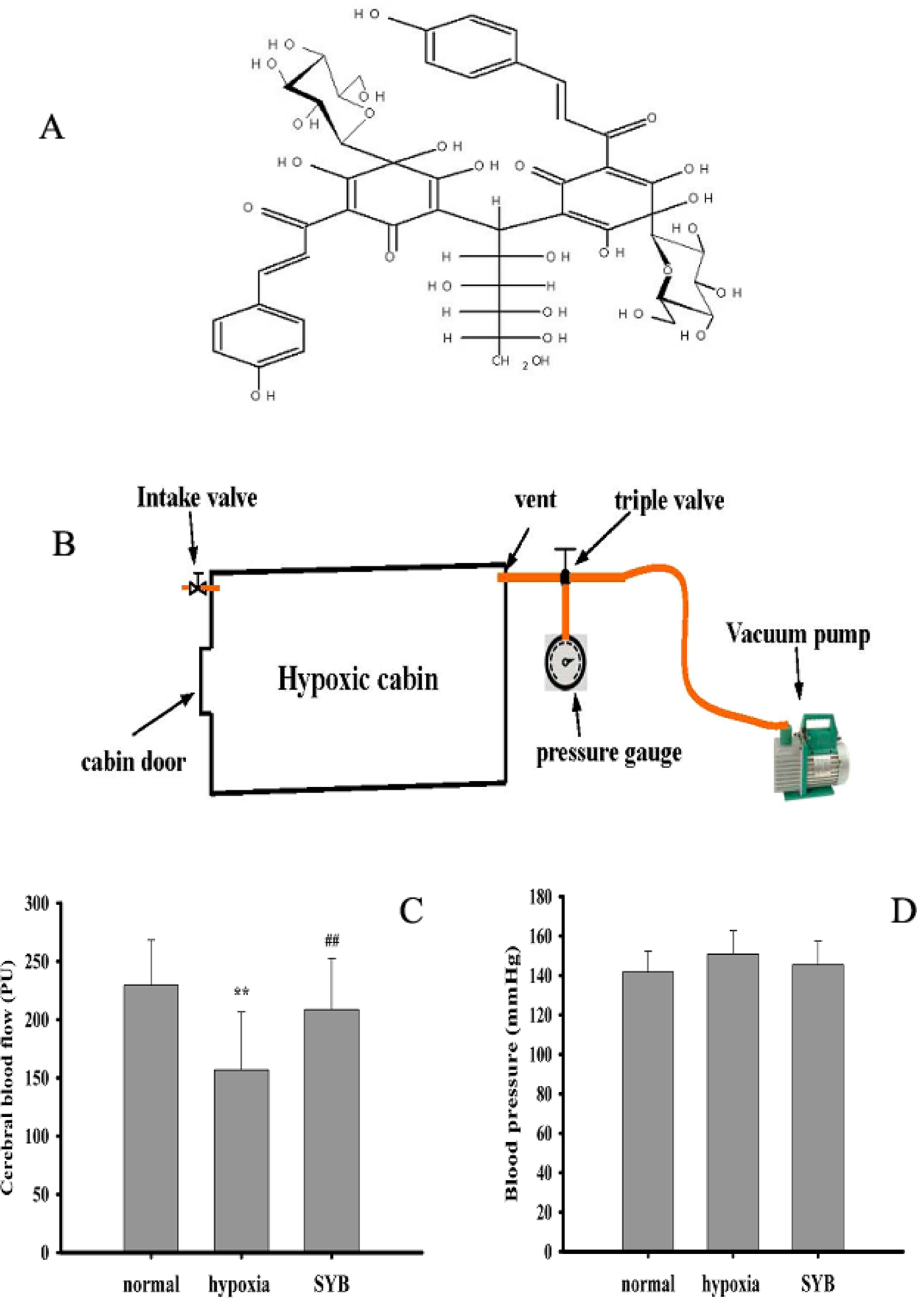
Effect of safflor yellow B on blood pressure and cerebral blood flow (**A**) Molecular structure of safflor yellow B (SYB). Figure 1B structure diagram of hypoxic cabin. As shown in (**B**–**D**) male SD rats were divided into three groups: normal, hypoxia, SYB. Except for normal group. all rats in other groups were reared in a temperature-controlled hypoxic cabin (Figure. 1B) with 314 mmHg and 46% oxygen for 4 weeks. SYB and saline were administered to rats in SYB and hypoxia group via intravenous injection using 2ML4 Alzet osmotic pumps, respectively. After 4 weeks, Cerebral blood flow (CBF) was monitored throughout using laser-Doppler flowmetry (LDF) (C) . Blood pressure was measured in anesthetized rats by the tail cuff method using the ALC-NIBP noninvasive blood pressure measurement (D). Data were presented as mean ± S.D. (*n* = 8). One-way ANOVA test was used to determine statistical significance. ^**^P < 0.01 vs. normal group, ^##^*P* < 0.01 vs. hypoxia group.

Safflor yellow B (SYB, Figure 1A) is a natural chalcone compound extracted from the safflower plant, *Carthamus tinctorius* L. (Compositae), which has been used extensively for the treatment of cardiovascular diseases in traditional Chinese medicine [21]. We previously reported that SYB increased antioxidant enzyme activities, reduced ROS levels, and alleviated vasoconstrictor factor-angiotensin II-induced EC injury [22, 23]. Recently, we found that SYB relaxed blood vessels and enhanced CBF. The present study aimed to investigate the involvement of the endothelium in SYB-mediated improvement in CBF and determine whether miRNAs and eNOS/NO signaling regulate this effect.

## RESULTS

### Effect of SYB on CBF and blood pressure

Hypoxia can reduce blood flow by enhancing vascular tension [7–9]. As shown in Figure 1C and 1D, compared to the normal group, long-term hypoxia significantly decreased CBF (*P* < 0.01), which was effectively reversed by SYB. However, the blood pressure did not differ significantly between these study groups (*P* > 0.05).

### Effect of SYB on apoptosis and cell viability

**Figure 2:**
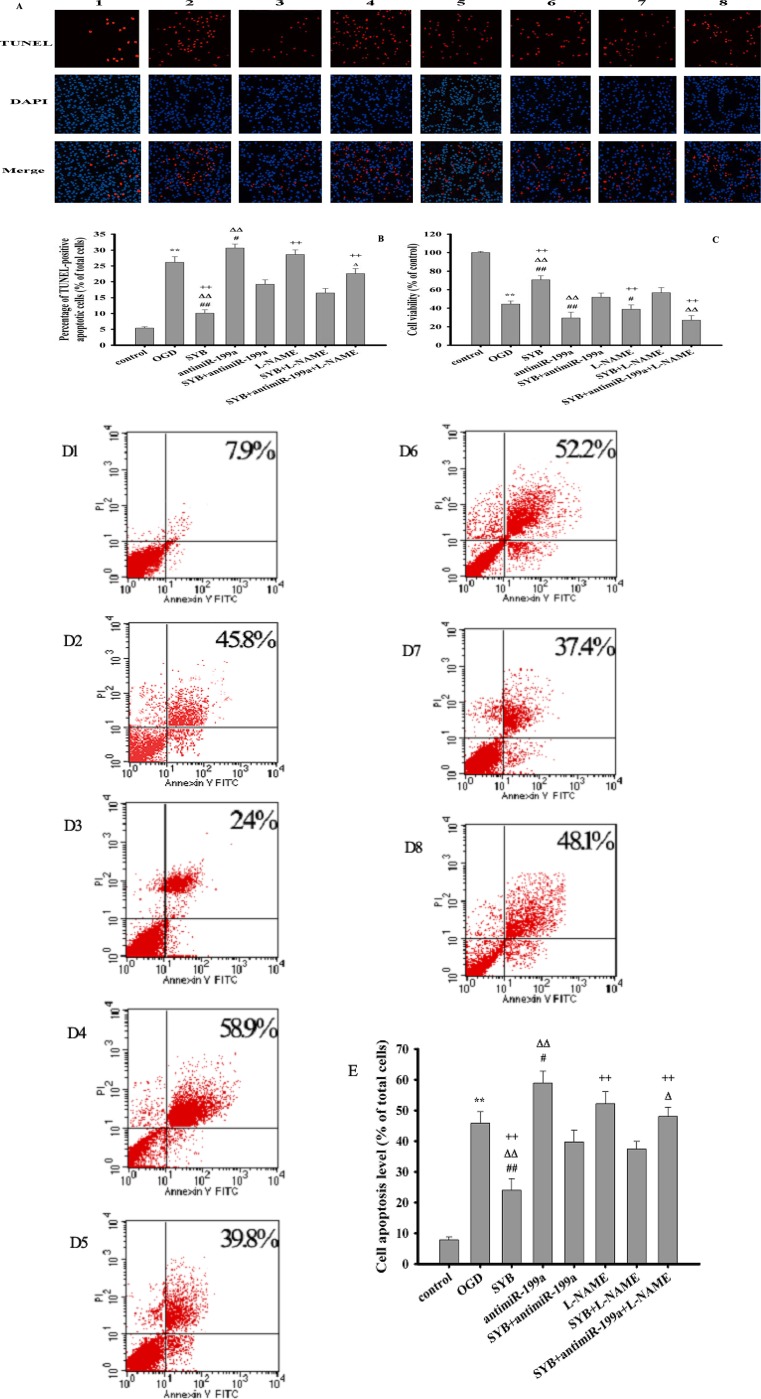
Effect of safflor yellow B on apoptosis and cell viability Rat aortic endothelial cells (RAECs) were cultured and divided into eight group: control group, OGD group, antimiR-199a group, L-NAME group, SYB group, antimiR-199a+SYBgroup, L-NAME +SYB group and antimiR-199a + L-NAME +SYB group. Except for normal group. all cells in other groups were treated with oxygen glucose deprivation (OGD) for 24 h. Apoptosis of all groups were observed by applying TUNEL apoptosis assay kit and AnnexinV/PI apoptosis assay kit, respectively. (**A**) apoptotic cells were visualized by TUNEL staining (red). Nuclei were counterstained with 4′,6-diamidino-2-phenylindole (DAPI, blue) ,scale bar indicates 100 μm(1,2,3,4,5,6,7,8 represent control group, OGD group, SYB group, antimiR-199a group, antimiR-199a+SYB group, L-NAME group, L-NAME+SYB group and antimiR-199a+L-NAME+SYB group respectively). (**B**) TUNELpositive apoptotic cells were counted as a percentage of the total number of cells. (**C**) Cell viabilities of all groups were measured by using MTT methods(Figure.2C). (**D**) cell activities were evaluated using AnnexinV/PI apoptosis assay kit (D1,D2,D3,D4,D5,D6,D7,D8 represent control group, OGD group, SYB group, antimiR-199a group, antimiR-199a+SYB group, L-NAME group, L-NAME+SYB group and antimiR-199a+L-NAME+SYB group respectively).E: Apoptotic cells obtained from AnnexinV/PI method were counted as a percentage of the total number of cells . Data were presented as mean ± S.D. (*n* = 3 for apoptosis, *n* = 8 for cell viability). One-way ANOVA test was used to determine statistical significance. ^**^P < 0.01 vs. normal group or control group, ^#^*P* < 0.05 or ^##^*P* < 0.01 vs. hypoxia group or OGD group, ^∆^*P* < 0.05 or ^D∆^*P* < 0.01 vs. SYB+antimiR-199a, ^+^*P* < 0.05 or ^++^*P* < 0.01 vs. L-NAME+SYB.

High NO levels, which are generated by the activation of iNOS, subsequently promoting the production of the reactive oxygen species (ROS) peroxynitrite (ONOO^-^), which causes apoptosis and cell injury [1–3]. As shown in Figure 2A, 2B, 2D and 2E, compared to the untreated control cells, oxygen-glucose deprivation (OGD) significantly increased the apoptosis of exposed cells (*P* < 0.01). Furthermore, this effect was enhanced by antimiR-199a (*P* < 0.05) and alleviated by SYB (*P* < 0.01). SYB effectively suppressed antimiR-199a- and N(G)-nitro-L-arginine methyl ester hydrochloride (L-NAME)-mediated apoptosis (*P* < 0.01). As shown in Figure 2C, OGD markedly reduced cell viability (*P* < 0.01) while SYB significantly increased cell viability and attenuated OGD-mediated cell injury (*P* < 0.01). Pre-treatment with antimiR-199a and L-NAME markedly enhanced the inhibitory effects of OGD on cell viability (*P* < 0.01 and *P* < 0.05, respectively). Moreover, these effects were reversed by SYB (*P* < 0.01). In addition, antimiR-199a significantly enhanced the L-NAME-mediated cell apoptosis and decreased the cell viability under OGD conditions (*P* < 0.01) while L-NAME comparably enhanced the antimiR-199a-induced cell injury (*P* < 0.05).

### Effect of SYB on ET-1-induced aortic contraction

**Figure 3:**
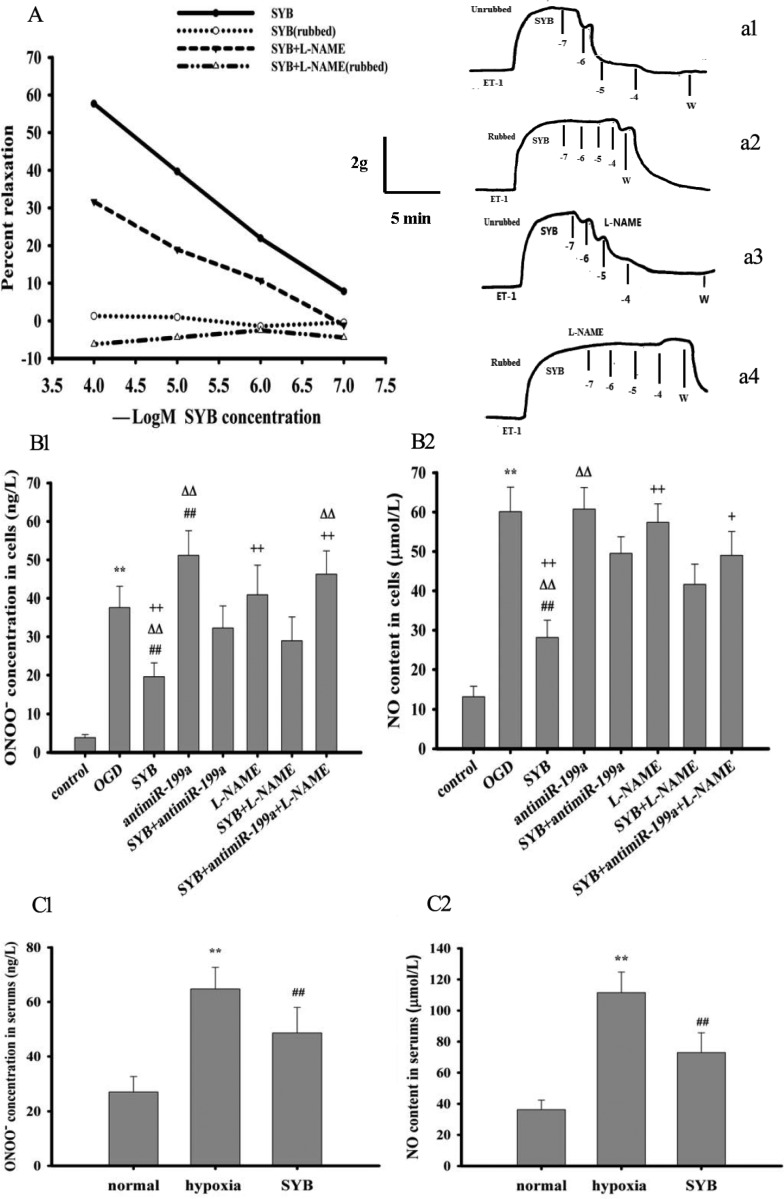
Effect of safflor yellow B (SYB) on aorta strips tension , ONOO^-^ and NO generation (**A**) Effect of SYB on aorta strips tension. As shown in (A) aorta strips were treated with with endothelin-1(ET-1) and ET-1+ L-NAME(500 nM) respectively. The vasorelaxant effect of different concentrations of SYB (10^−7^–10^−4^ M) on the contraction induced by ET-1 was evaluated (a1: endothelium unrubbed; a2: endothelium rubbed; a3:L-NAME with endothelium; a4: L-NAME without endothelium). (**B**, **C**) Effect of SYB on ONOO^-^ and NO generation. Rats were divided into three groups. Except for normal group. the rats in hypoxia group and SYB group were reared in a temperature-controlled hypoxic cabin for 4 weeks. Rat aortic endothelial cells (RAECs) were cultured and divided into eight group: control group, OGD group, antimiR-199a group, L-NAME group, SYB group, antimiR-199a+SYBgroup, L-NAME +SYB group and antimiR-199a + L-NAME +SYB group. As shown in (**B1**, **B2**, **C1** and **C2**) the levels of ONOO¯ and NO in serum and RAECs were detected in according to the procedure described as ELISA kits, respectively. Data were presented as mean ± S.D. (*n* = 8). One-way ANOVA test was used to determine statistical significance. ^**^P < 0.01 vs. normal group or control group, ^##^*P* < 0.01 vs. hypoxia group or OGD group, ^D∆^*P* < 0.01 vs. SYB+antimiR-199a, ^+^*P* < 0.05 or ^++^*P* < 0.01 vs. L-NAME+SYB.

ET-1 regulates vasoconstriction and blood flow to target organs [10, 24], but eNOS in ECs plays a key role in maintaining local blood flow to organs [5, 6]. As shown in Figure 3A, ET-1 significantly increased the contractile tension of aortic strip preparations while SYB effectively suppressed this effect and produced a relaxant effect (a1). However, pre-treatment with L-NAME markedly enhanced the contractile effect of ET-1 on aortic strips and attenuated the SYB-mediated relaxation (a3). After removing the endothelium (a2 and a4), the ET-1-induced contraction of the aortic strips was elevated, and this effect was strengthened by L-NAME while the relaxant effect of SYB was markedly reduced. Interestingly, the effect of SYB was further attenuated by treatment with L-NAME.

### Effect of SYB on ONOO^-^ and NO levels

*In vivo* and *in vitro* experiments confirmed that hypoxia increased inducible NOS (iNOS) expression and enhanced the generation of NO, which reacted with ROS to form ONOO^-^ (Figure 3B1-C2). The present study also showed that SYB effectively reduced the levels of NO and ONOO^−^ in serum and rat aortic ECs (RAECs, *P* < 0.01). Pre-treatment of RAECs with antimiR-199a significantly elevated the ONOO^-^ level compared with that of cells exposed to OGD only (*P* < 0.01), while this effect was significantly alleviated by SYB (*P* < 0.01). Compared with the control cells, L-NAME-treated cells did not exhibit enhanced levels of NO or ONOO^-^. Furthermore, L-NAME or antimiR-199a effectively suppressed the inhibitory effects of antimiR-199a +SYB or L-NAME + SYB on ONOO^-^ overproduction (*P* < 0.01) while antimiR-199a markedly attenuated the effect of L-NAME + SYB on NO generation (*P* < 0.05).

### Effect of SYB on protein kinase C (PKC) activity

**Figure 4:**
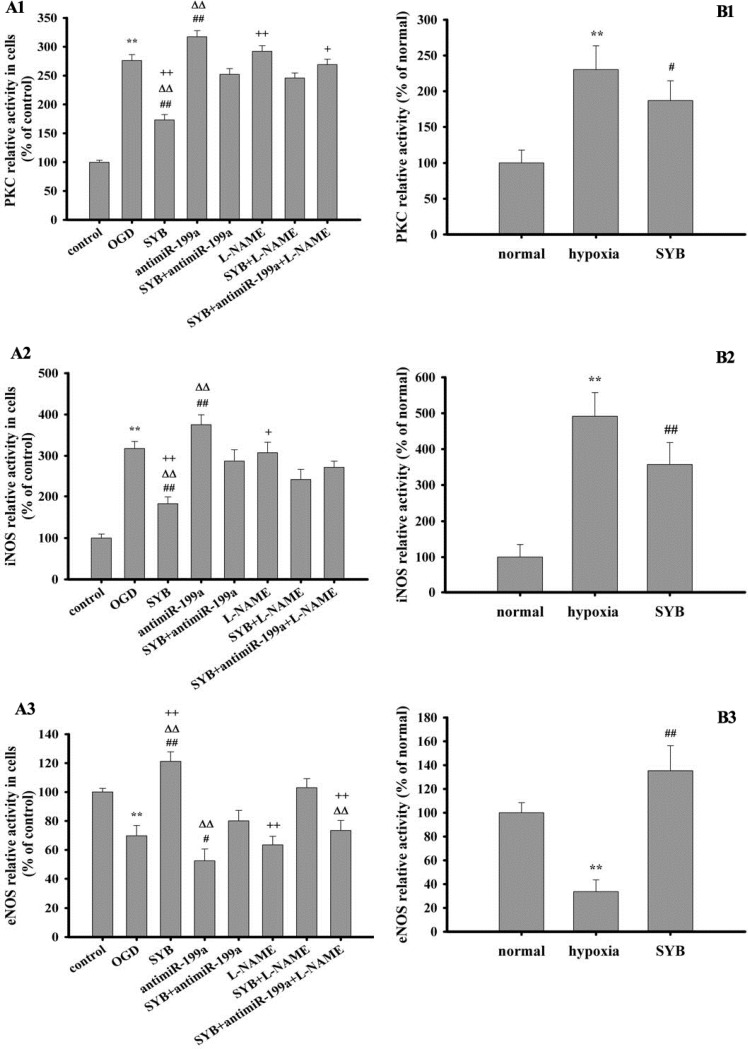
Effect of safflor yellow B (SYB) on the activities of protein kinase C (PKC), inducible nitric oxide synthase (iNOS) and endothelial nitric oxide synthase (eNOS) (**A1**, **A2**, **A3**) Effect of safflor yellow B (SYB) on the activities of PKC, iNOS and eNOS *in vitro.* RAECs were used to establish OGD model. RAECs were homogenized and centrifuged at 4°C, and the supernatants were used to measure the activities of PKC, iNOS and eNOS according to the procedure described as assay Kits. A1, A2, A3 represent the activities of PKC, iNOS and eNOS in RAECs. (**B1**, **B2**, **B3**) Effect of safflor yellow B (SYB) on the activities of PKC, iNOS and eNOS *in vivo*. Rats were divided into three groups: normal, hypoxia, SYB. Except for normal group. all rats in other groups were reared in hypoxic cabin. The serum and vascular endotheliums were obtained aftet 4 weeks. B1 represents PKC activity in vascular endotheliums, and B2 and B3 represent the activities of iNOS and eNOS in serums. Data were presented as mean ± S.D. (*n* = 8 in tissues or serums, *n* = 3 in cells). One-way ANOVA test was used to determine statistical significance. ^**^P < 0.01 vs. normal group or control group, ^#^*P* < 0.05 or ^##^*P* < 0.01 vs. hypoxia group or OGD group, ^D∆^*P* < 0.01 vs. SYB+ antimiR-199a, ^+^*P*< 0.05 or ^++^*P* < 0.01 vs. L-NAME+SYB.

Exposure to hypoxia or OGD significantly increased PKC activity in the vascular endothelium and RAECs (*P* < 0.01) compared with that in the respective controls (Figure 4A1 and B1). Compared to hypoxic and OGD conditions, SYB treatment remarkably decreased the PKC activity (*P* < 0.05 and *P* < 0.01, respectively). In cells exposed to OGD, antimiR-199a significantly enhanced PKC activity (*P* < 0.01), but this effect was not observed in the presence of L-NAME. SYB markedly reduced PKC activity compared with OGD + antimiR-199a or OGD + L-NAME (*P* < 0.01). Under OGD conditions, antimiR-199a more effectively alleviated the inhibitory effect of SYB on PKC than L-NAME did. Furthermore, SYB + antimiR-199a +L-NAME was more effective than SYB+ antimiR-199a or SYB+ L-NAME was.

### Effect of SYB on iNOS and eNOS activities

Exposure to hypoxia or OGD significantly promoted iNOS activity in the serum or RAECs (*P* < 0.01) and reduced eNOS activity (*P* < 0.01) compared with their respective controls (Figure 4A2–B3). SYB attenuated the effects of hypoxia and OGD on iNOS and eNOS (*P* < 0.01). AntimiR-199a markedly increased iNOS activity and reduced eNOS activity compared with that of cells exposed to OGD only (*P* < 0.01 and *P* < 0.05), and these effects were remarkably reversed by SYB (*P* < 0.01). The activities of iNOS and eNOS did not differ significantly between the L-NAME and OGD groups, while they were significantly affected by SYB (*P* < 0.01 or *P* < 0.05). L-NAME and anti-miR-199a effectively alleviated the effects of SYB on iNOS and eNOS in cells exposed to OGD (*P* < 0.01 and *P* < 0.05, respectively).

### Effect of SYB on ET-1 generation

**Figure 5:**
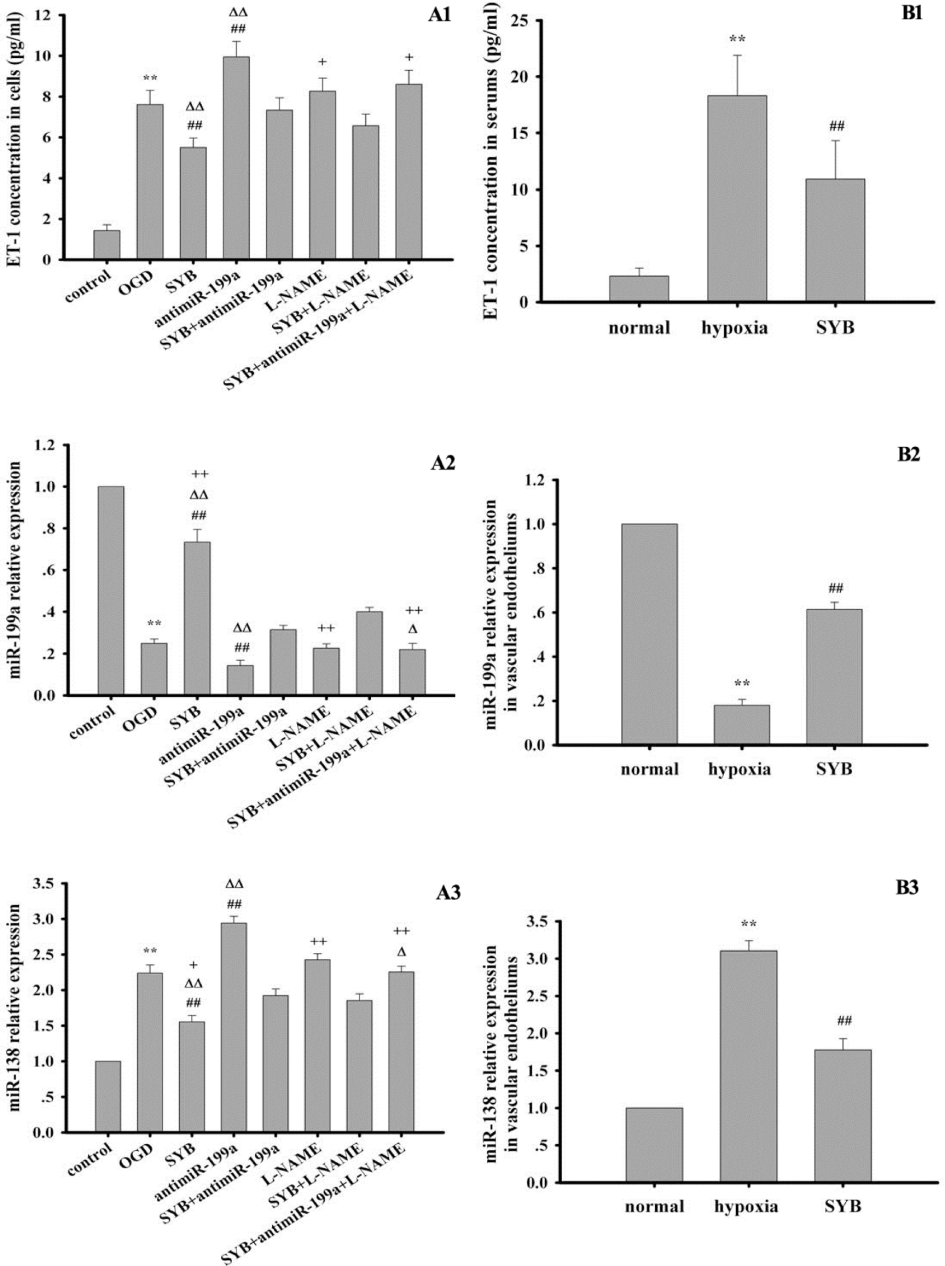
Effect of safflor yellow B (SYB) on endothelin-1 (ET-1) content and the expression levels of miR-199a and miR-138 (**A1** and **B1**) Effect of SYB on ET-1 content. Rats and RAECs were used to establish hypoxia model and OGD model respectively. The supernatants from RAECs and serums were used to measure ET-1 content according to the instructions described as ELISA kits.A1and B1 represent ET-1 content in RAECs and serums respectively. (**A2**–**B3**) Effect of SYB on the expression levels of miR-199a and miR-138. Total RNA were isolated from vascular endotheliums and RAECs using Trizol (Invitrogen) according to manufacturer’s instruction. cDNA was synthesized using miScript reverse transcription kit. Relative expression of miR199a and miR-138 was calculated using the 2 ^−ΔΔCT^ method on a real-time PCR system. A2, B2, A3 and B3 represent the expression levels of miR-199a and miR-138 in RAECs and vascular endotheliums respectively. Data were presented as mean ± S.D. (*n* = 3). One-way ANOVA test was used to determine statistical significance. ^**^P < 0.01 vs. normal group or control group, ^##^*P* < 0.01 vs. hypoxia group or OGD group, ^∆^*P* < 0.05 or ^D∆^*P* < 0.01 vs. SYB+antimiR-199a, ^+^*P* < 0.05 or ^++^*P* < 0.01 vs. L-NAME+SYB.

As shown in Figure 5A1–B1, hypoxia and OGD significantly enhanced the levels of ET-1 in the serum or RAECs, and this effect was enhanced by antimiR-199a (*P* < 0.01). SYB obviously reduced the increase in ET-1 induced by hypoxia and OGD (*P* < 0.01), which were markedly attenuated after treatment with antimiR-199a. Although L-NAME tended to enhance ET-1 generation, no significant differences occurred between the L-NAME and the OGD groups. In cells exposed to OGD and SYB, co-treatment with L-NAME and antimiR-199a further elevated the ET-1 levels (*P* < 0.05) compared to L-NAME treatment alone.

### Effect of SYB on miR-199a and miR-138 expression

Hypoxia and OGD evidently reduced and increased miR-199a and miR-138 expression levels, respectively in the vascular endothelium or RAECs compared with the respective controls (Figure 5B2–C3). Moreover, these effects were ameliorated by SYB (*P* < 0.01). Treatment with antimiR-199a downregulated miR-199a and upregulated miR-138 (*P* < 0.01) while SYB significantly attenuated these effects in RAECs (*P* < 0.01). In cells exposed to OGD, L-NAME did not significantly affect the expression of miR-199a and miR-138 while their expression levels were upregulated and downregulated, respectively following treatment with SYB and L-NAME.

### Effect of SYB on target protein expression

**Figure 6:**
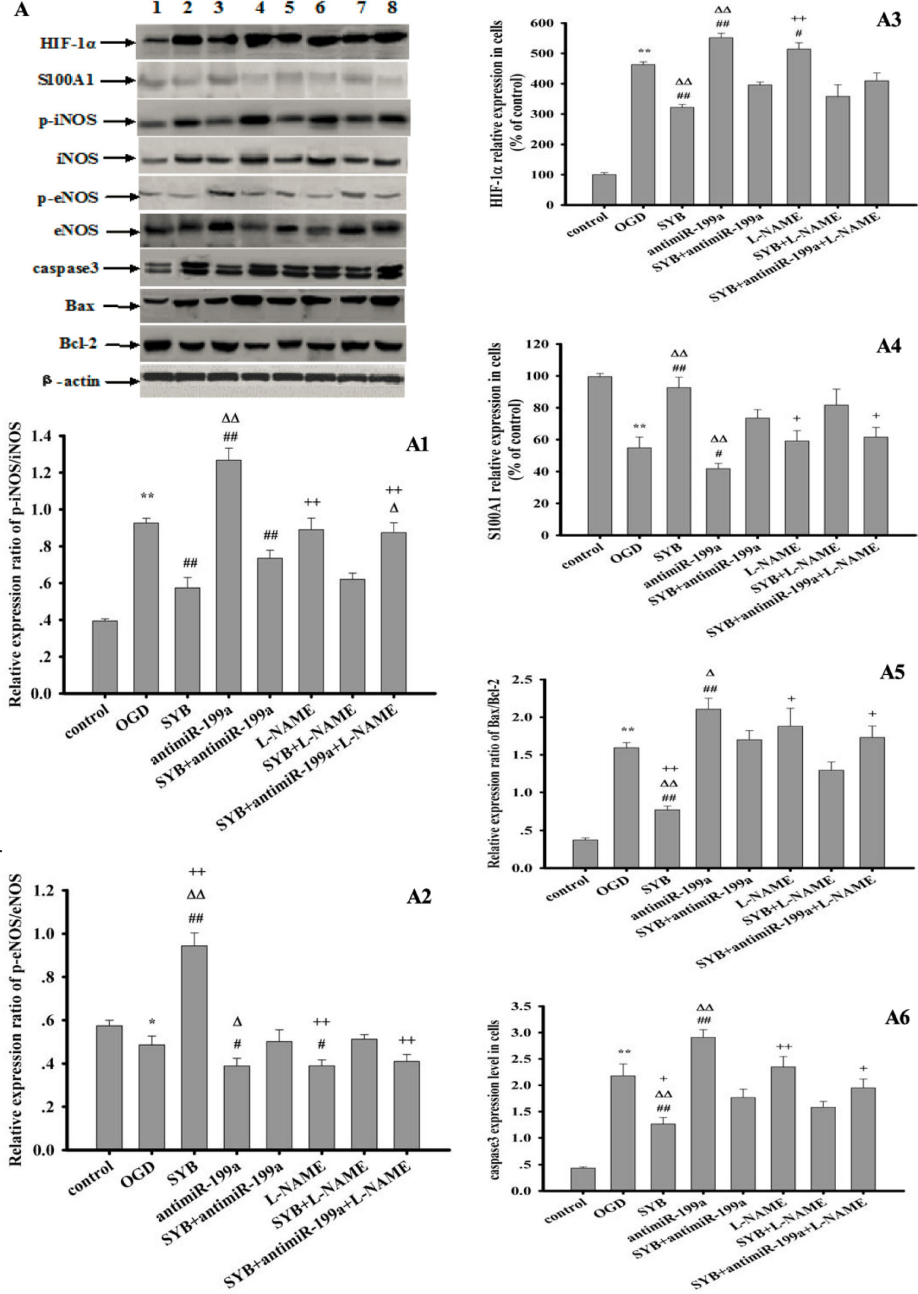
Effect of safflor yellow B (SYB) on target protein expression in RAECs RAECs were used to establish OGD model. Total proteins in RAECs from different groups were extracted and measured. Thirty micrograms of total proteins were loaded per lane, and separated by SDS-PAGE and transferred to PVDF membrane. After incubation with secondary antibodies, the expression levels of HIF-1α, S100A1, p-iNOS, iNOS, p-eNOS, eNOS, caspase3,Bax, Bcl-2 and β-actin were visualized using chemiluminescence method. A represent Western blots in panel (1, 2, 3, 4, 5, 6, 7, 8 represent control group, OGD group, SYB group, antimiR-199a group, antimiR-199a+SYB group, L-NAME group, L-NAME+SYB group and antimiR-199a+L-NAME+SYB group respectively). (**A1**, **A2**, **A3**, **A4**, **A5**, **A6)** represent the relative ratio of p-iNOS/iNOS and p-eNOS/eNOS, HIF-1α, S100A1,relative ratio of Bax/Bcl-2, caspase3 respectively. Data were presented as mean ± S.D. (*n* = 3). One-way ANOVA test was used to determine statistical significance. *P < 0.05 or^**^P < 0.01 vs. control group, ^#^*P* < 0.05 or ^##^*P* < 0.01 vs. OGD group, ^∆^*P* < 0.05 or ^D∆^*P* < 0.01 vs. SYB+antimiR-199a, ^+^*P*< 0.05 or ^++^*P* < 0.01 vs. L-NAME+SYB.

**Figure 7:**
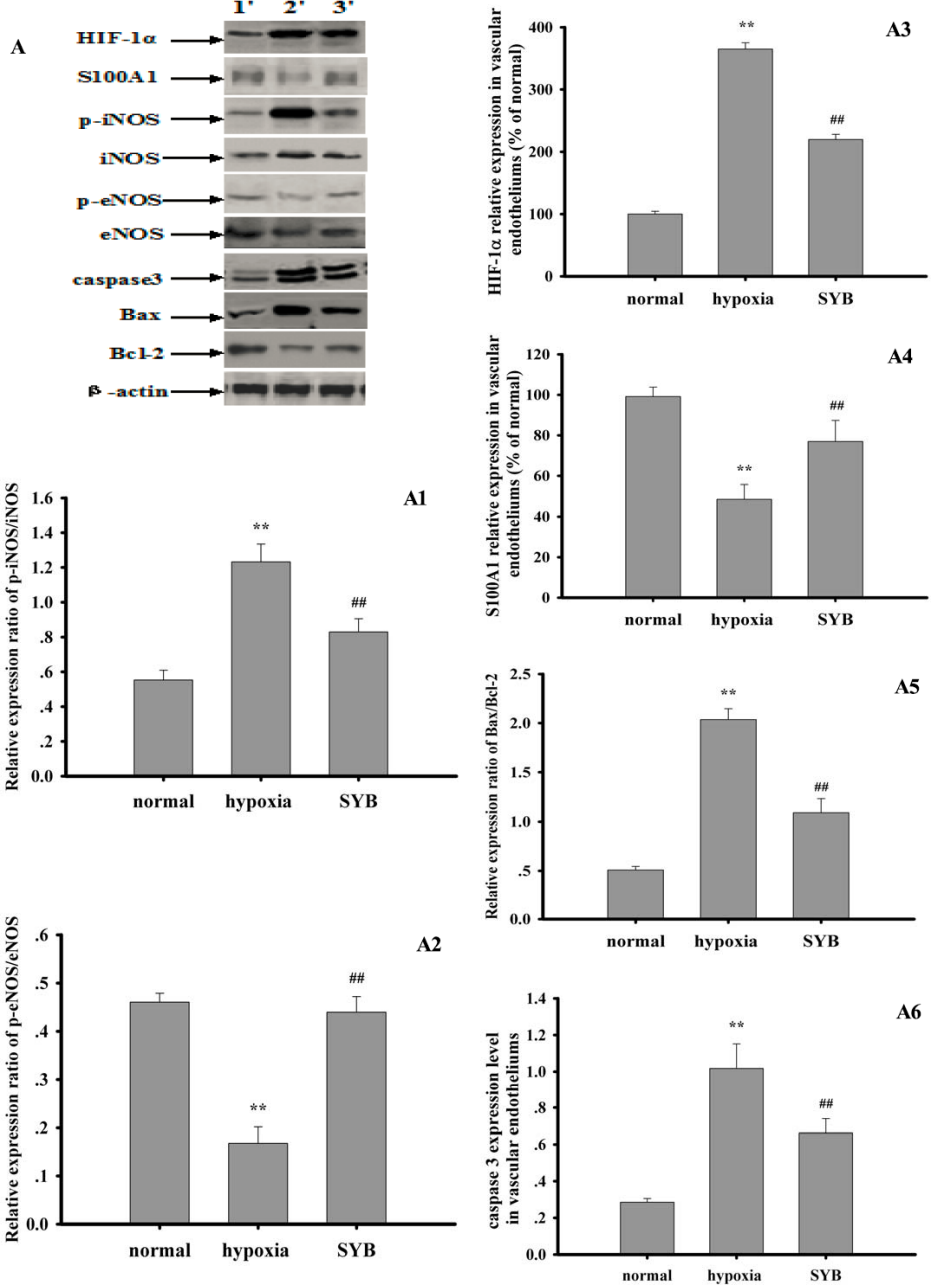
Effect of safflor yellow B on target protein expression in vascular endotheliums Rats were used to establish hypoxia model. The vascular endotheliums of thoracic aorta were obtained by using cell scraper to gently scratch. Total protein in RAECs from different groups were extracted and measured. Thirty micrograms of total proteins were loaded per lane, and separated by SDS-PAGE and transferred to PVDF membrane. After incubation with secondary antibodies, the expression levels of HIF-1α, S100A1, p-iNOS, iNOS, p-eNOS, eNOS, caspase3, Bax, Bcl-2 and β-actin were visualized using chemiluminescence method. A. represents Western blots in panel (1, 2, 3 represent normal group, hypoxia group, SYB group respectively). **A1**, **A2**, **A3**, **A4**, **A5**, and **A6** represent the relative ratio of p-iNOS/iNOS and p-eNOS/eNOS, HIF-1α, S100A1,relative ratio of Bax/Bcl-2, caspase3 respectively. Data were presented as mean ± S.D. (*n* = 3). One-way ANOVA test was used to determine statistical significance. **P < 0.01 vs. normal group, ##*P* < 0.01 vs. hypoxia group.

As shown in Figures 6 and 7, exposure to hypoxia or OGD significantly altered target protein levels in the vascular endothelium or RAECs. Furthermore, these studies detected increased expression levels of HIF-1α and caspase 3, increased B-cell lymphoma-2 (Bcl-2)-associated X protein (Bax)/Bcl-2 and phosphorylated iNOS at Tyr 151 (p-iNOS^Tyr151^)/iNOS ratios, reduced S100A1 expression, and reduced the p-eNOS^Ser1177^/eNOS ratio. SYB attenuated the increase in HIF-1α and caspase 3 levels, reduced the Bax/Bcl-2 and p-iNOS^Tyr151^/ iNOS ratios, and maintained the levels of eNOS, p-eNOS^Ser1177^, and S100A1. After blocking miR-199a expression, the effects of OGD on target protein expression in RAECs significantly increased, as indicated by the greater inhibition of eNOS, p-eNOS^Ser1177^, Bcl-2, and S100A1, and greater promotion of HIF-1α, Bax, caspase 3, iNOS, and p-iNOS^Tyr151^ (*P* < 0.01 or *P* < 0.05). AntimiR-199a attenuated the SYB-mediated elevation of the p-eNOS^Ser1177^/eNOS ratio and S100A1 expression (*P* < 0.01) and reversed the inhibitory effects of SYB on HIF-1α, caspase 3, and Bax/Bcl-2 and p-iNOS^Tyr151^/iNOS (*P* < 0.01) ratios. L-NAME significantly increased HIF-1α levels and reduced the p-eNOS^Ser1177^/eNOS ratio compared with that of cells exposed to OGD only (*P* < 0.01). Following the blockade of eNOS activity by L-NAME, SYB significantly reduced HIF-1α and caspase 3 levels, suppressed the p-iNOS^Tyr151^/iNOS and Bax/Bcl-2 ratios, and enhanced both S100A1 expression and the p-eNOS^Ser1177^/eNOS ratio (*P* < 0.01 or *P* < 0.05).

## DISCUSSION

NO is a molecular messenger that plays important roles in the regulation of cell proliferation, differentiation, and activity and is also recognized as a critical contributor to the development of atherosclerosis and cardiovascular disease [25]. Basal (low) concentrations of NO are generated by eNOS and neuronal NOS and are essential for physiological functions, which can both improve endothelial function and reduce ROS levels [26, 27]. High NO levels are generated by iNOS in response to bacterial components and proinflammatory cytokines and are essential for immune functions by stimulating HIF-1 activity and increasing ONOO^-^ generation [1, 3]. In the present study, exposure to hypoxia (*in vivo*) and OGD (*in vitro*) significantly increased NO levels in the serum and RAECs, respectively. HIF-1α levels and iNOS activation also increased, resulting in ONOO^−^ generation and reduced cell viability. SYB effectively reduced NO production by downregulating HIF-1α and inhibiting iNOS.

Low oxygen environments increase the expression of HIF-1α, the major hypoxia-regulated transcription factor [28]. HIF-1α binds to the hypoxia-response element and affects the activation and expression of PKC [29]. PKC also directly stimulates HIF-1 expression [30] and increases iNOS activity [31] and indirectly decreases eNOS activity [32].

The present study showed that hypoxia or OGD significantly enhanced HIF-1α expression and stimulated PKC activation and, thus promoted the levels of iNOS and p-iNOS^Tyr151^ in the vascular endothelium or RAECs. eNOS expression and function, which play critical roles in regulating blood vessel wall function and maintaining local blood flow, have been implicated in the pathology of a number of cardiovascular diseases including atherosclerosis and hypertension [1, 6]. Furthermore, loss of eNOS expression or activity is a key defining feature of clinical endothelial dysfunction. Consistent with previous studies [14, 33], we found that hypoxia or OGD significantly downregulated the expression of eNOS and p-eNOS^Ser1177^ in the vascular endothelium or RAECs. This indicates that hypoxia and OGD promoted iNOS activation and eNOS inhibition by upregulating HIF-1α expression and stimulating PKC activity.

Furthermore, these effects compromised the endothelial function and, thereby, significantly reduced CBF and RAEC viability. L-NAME is a NOS inhibitor with a considerably lower half-maximal inhibitory concentration (IC_50_) against eNOS (500 nM) than that against iNOS. In this study, we selectively inhibited eNOS activity by using L-NAME at an appropriate concentration (500 nM). After treatment with L-NAME at 500 nM, eNOS was inhibited, and NO was generated by the OGD-induced activation of iNOS. This action further improved the stability and activation of HIF-1α [13, 34], leading to ONOO^-^ generation and apoptosis of ECs. SYB effectively increased eNOS activity by downregulating the HIF-1α/PKA signaling pathway, promoting endothelial NO generation, and reducing ONOO^-^ generation. These actions improved the vascular function and increased CBF, and were attenuated by L-NAME.

Ischemia can stimulate brain tissue to synthesize ROS, which promote cytochrome c release from the mitochondria, which stimulates caspase 3 and Bax expression and inhibits Bcl-2 expression, leading to apoptosis [35, 36]. Consistent with these previous reports, the present study revealed that hypoxia or OGD triggered ONOO^-^ generation and increased the levels of caspase 3 and Bax. SYB acted as an antioxidant by suppressing ONOO^−^ generation via upregulation of eNOS and downregulation of iNOS, which reduced caspase 3 expression, maintained the Bcl-2/Bax ratio, and inhibited apoptosis of RAECs.

ET-1 is involved in the development of hypertension and other cardiovascular diseases [37]. The ET-1 level regulates vasoconstriction and blood flow to target organs [8, 24]. Previous studies have indicated that hypoxia is a potent inducer of *ET-1* gene expression in ECs by activating HIF-1α [11, 12]. Consistent with this, the results of the present study revealed that exposure to hypoxia or OGD significantly enhanced ET-1 and HIF-1α levels, thereby reducing CBF *in vivo*. SYB effectively reduced the levels of ET-1 by inhibiting HIF-1α, consequently increasing the CBF.

miRNAs have been identified as major factors in numerous aspects of vascular homeostasis and the pathogenesis of cardiovascular diseases including angiogenesis, vascular remodeling, and myocardial infarction [38]. miR-199a affects HIF-1α mRNA translation by binding to the 3′ untranslated region [19, 20]. The overexpression of miR-199a was shown to reduce hypoxia-induced HIF-1α expression and suppress iNOS activity [19, 20]. The present study also showed that hypoxia or OGD reduced miR-199a expression and, thereby, upregulated HIF-1α and increased iNOS expression. S100A1 augmented the enzymatic activity of eNOS in ECs by preventing PKC-mediated phosphorylation of t eNOS [18]. miR-138 directly suppresses S100A1 mRNA translation by binding to its 3′ untranslated region and, thus, indirectly attenuates eNOS activity in ECs by the loss of S100A1 [18]. Most et al. [18] reported that hypoxia significantly diminished S100A1 levels in ECs. In the vascular endothelium and RAECs, S100A1 was significantly downregulated by hypoxia- and OGD-induced overexpression of miR-138. SYB effectively downregulated miR-138 and, thereby, increased the S100A1 level. This effect significantly enhanced eNOS activity and alleviated hypoxia-induced endothelial dysfunction and OGD-mediated damage to RAECs.

Taken together, these results indicate that SYB attenuated hypoxia-induced vasoconstriction by upregulating miR-199a expression, decreasing HIF-1α expression, and activating the eNOS/NO signaling pathway.

## MATERIALS AND METHODS

### Animal model of hypoxia

Safflor yellow B (SYB, a yellow amorphous powder with a purity > 98% as determined by using high-performance liquid chromatography (HPLC), Figure 1A). Male Sprague-Dawley rats weighing 250–300 g were purchased from the Experimental Animal Department of Shandong Luye Pharmaceutical Co. Ltd., (Yantai, China) and randomly divided into three groups (*n* = 8 each): normal, hypoxia, and SYB (24 mg/kg). The rats in the normal group were fed at an altitude of 0 m on a 12-h light-dark cycle for 4 weeks. Rats in the hypoxia and SYB groups were maintained in a temperature-controlled hypoxic cabin (Figure 1B) on a 12-h light-dark cycle (light on at 06:00 AM) for 4 weeks. This mimicked the low-pressure, low-oxygen environment at an altitude of 7,000 meters (314 mmHg, oxygen content equivalent to 46% of that at sea level). SYB and saline were intravenously injected into rats in the SYB and hypoxia group, respectively using the 2ML4 Alzet osmotic pumps (DURECT Corp., Cupertino, CA, USA). All the animals were treated in accordance with the National Institutes of Health (NIH) Guide for the Care and Use of Laboratory Animals. The animal care and experimental procedures were approved by the Ethics Committee in Animal and Human Experimentation of Binzhou Medical University.

### Detection of CBF and blood pressure

All the rats were anesthetized with 10% chloral hydrate in 0.9% sodium chloride (NaCl) after 4 weeks, and their heads were fixed in a stereotactic holder while they were in a prone position. Under sterile conditions, a burr hole was drilled 3 mm posterior and 5 mm lateral to the bregma, leaving the dura intact. A small laser-Doppler flowmetry (LDF) probe (Perimed PF5001, Sweden) was placed on the dura, fixed to the skull with cyanoacrylate and dental cement, and used to monitor the CBF continuously. The blood pressure was measured in anesthetized rats by using the tail cuff method using the automatic level control (ALC)-noninvasive blood pressure (NIBP) measurement and analysis system (Alcott Biotech Co., Ltd., Shanghai, China). Each value was presented as the average of three independent readings.

### Preparation of isolated aortic strips

The rats were euthanized by decapitation under anesthesia, the abdominal aortas were collected from each study group, and stored at −80°C for molecular biological studies. Furthermore, the thoracic aortas of the normal group were rapidly dissected and placed in Krebs-bicarbonate solution (pH 7.4) consisting of 120 mM NaCl, 4.8 mM potassium chloride (KCl), 1.5 mM calcium chloride (CaCl_2_), 1.2 mM magnesium sulfate (MgSO_4_), 1.2 mM monopotassium phosphate (KH_2_PO_4_), 20 mM sodium bicarbonate (NaHCO_3_), and 10 mM d-glucose. After removing the excess fat and connective tissue, each aorta was cut into helical strips approximately 2–3 mm wide and 15–20 mm long, which were then mounted in 37°C organ baths containing 10 mL oxygenated Krebs buffer (95% O_2_, 5% CO_2_).

Each preparation was connected to a muscle tension transducer (JZ101, Gaobeidian Xinhang Electromechanical Equipment Co., Ltd., Hebei, China). Isometric tension was recorded using a chart software (MLS023, PowerLab, AD Instruments, Australia). The strips were mounted, allowed to stabilize for 1 h, and then gradually stretched to achieve an optimal resting tension of 1.0 g prior to treatment with ET-1 (5 nM, Sigma-Aldrich, Shanghai, P.R. China) or ET-1 and L-NAME (500 nM). The vasorelaxant effects of SYB (10^–7^–10^–4^ M) on the ET-1-induced contraction were then evaluated, and drug treatment effects were expressed as a percentage of the maximal strip contraction. To investigate the role of the endothelium, it was removed mechanically by gentle rubbing with a cotton bud. The vasorelaxant effects of SYB on ET-1- and L-NAME-induced contractions were measured in the endothelium-denuded preparations [39]. For these studies, the vasodilation was expressed as a percentage of the ET-1-induced contraction.

### Cell culture

RAECs were isolated and cultured as previously described with minor modifications [40, 41]. Segments of the thoracic aorta were removed from Sprague-Dawley rats (180–200 g) and immediately placed in cold phosphate-buffered saline (PBS) containing 100 U/mL penicillin and 100 mg/mL streptomycin. The aorta was gently cleaned to remove the periadventitial fat and connective tissue and cut into 5 mm × 5 mm flat segments, which were placed endothelial side down in a dish with medium 199 containing 20% fetal bovine serum, 2.5 ng/mL basic fibroblast growth factor, 100 U/mL penicillin, and 100 mg/mL streptomycin. The aortic segments were cultured at 37°C in an atmosphere of 95% air/5% CO_2_ for 72–80 h without movement and were removed when the cells had migrated.

The cytological characteristics of the RAECs were investigated by using acetylated low-density lipoprotein (LDL) labeled with 1,1′-dioctadecyl-3,3,3′,3′-tetramethyl-indocarbocyanine perchlorate (DiI-Ac-LDL, Molecular probe, Eugene, OR, USA) staining, as described previously [42]. All experiments were performed with RAECs at passage number ≤ 4, which were seeded in six- or 96-well plates. The cells were divided into eight treatment groups: control, OGD, antimiR-199a, L-NAME, SYB, antimiR-199a + SYB, L-NAME + SYB, and antimiR-199a + L-NAME + SYB groups. All the cells (except for the normal group) were treated with the drugs or inhibitors in the culture media for 24 h prior to a 24-h exposure to OGD. AntimiR-199a (50 nM) was added in O-minimum essential medium (MEM, Gibco,invitrongen,USA) with Lipofectamine^™^ 2000 (Invitrogen). For the single treatments, L-NAME (500 nM) or SYB (0.8 mM) was added to the O-MEM. For combined treatments, antimiR-199a (50 nM), L-NAME (0.5 μM), SYB (0.8 mM), or a combination of these agents was used. The same volume of the vehicle was added to the control group cells.

### Cell viability and apoptosis assays

The 3-(4,5-dimethyithiazol-2-yl)-2,5-diphenyl-tetrazolium bromide (MTT) assay was used to determine cellular mitochondrial dehydrogenase activity in primary cortical cells cultured in 96-well plates, as described in the “cell culture” section. The dark blue formazan crystals formed in the intact cells were solubilized with dimethyl sulfoxide (DMSO), and the absorbance was measured at 490 nm using a microplate reader (BioTek Synergy H4, USA). The results were expressed as a percentage of MTT reduction in the vehicle-treated control cells with an assumed absorbance of 100%.

Cell apoptosis were detected by using the terminal deoxyribonucleotidyl transferase-mediated dUTP-digoxigenin nick-end labeling (TUNEL) assay and AnnexinV-FITC/Propidium iodide (PI) apoptosis assay methods, respectively. Briefly, primary RAECs were cultured and treated according to the instructions provided in the TUNEL and AnnexinV-FITC/PI apoptosis assay kits (Beyotime Institute of Biotechnology, China). The nuclei were labeled using 4′,6-diamidino-2- phenylindole (DAPI, Leagene Biotech, Beijing, China) and the active and apoptotic cells (blue and red, respectively) were visualized using an IX70-inverted fluorescence microscope (Olympus, Tokyo, Japan). TUNEL-positive apoptotic cells were expressed as a percentage of the total number of cells.The cells labeled AnnexinV /PI were then examined by flow cytometry (Beckman Coulter Inc., Brea, CA, USA), and the percentage of the viable apoptotic cell was calculated. Independent experiments were repeated at least three times.

### Measurement of ONOO^-^ and NO levels

Rat serum was obtained by centrifugation of blood at 3,000 *g* for 15 min at 4°C. RAECs were collected and lysed using ultrasound before centrifugation at 12,000 *g* for 5 min at 4°C. The levels of ONOO^-^ and NO in the serum and cell supernatant samples were analyzed using enzyme-linked immunosorbent assay (ELISA) kits (Shanghai Yueyan Biological Technology Co., Ltd., China and Nanjing Jiancheng Biology Engineering Institute, China, respectively) according to the manufacturers’ instructions. For the ONOO^−^ assay, 50 μL assay mixture was added to each well containing the ONOO^−^ standards, blank control, or samples to achieve a total assay volume of 100 µL/well. Following incubation at 37°C for 30 min in the dark, the optical density at 450 nm was measured using a BioTek Synergy H4 microplate reader (BioTek Synergy H4, USA). ONOO^−^ concentrations were expressed as nanograms per liter (ng/L), and NO levels were expressed as micromoles per liter (μmol/L).

### Determination of iNOS and eNOS activities

The activities of iNOS and eNOS were measured in rat serum and RAEC supernatant samples using iNOS and eNOS ELISA kits (CUSBIO, China) according to the manufacturer’s instructions. Briefly, 100 μL sample was incubated with 100 μL biotinylated antibody at 37°C for 1 h prior to washing thrice and incubating with horseradish peroxidase (HRP)-avidin for 1 h at 37°C. Finally, 90 μL tetramethylbenzidine substrate was added and the optical density of the resultant solution was detected at 450 nm using a BioTek Synergy H4 microplate reader.

### Detection of ET-1

ET-1 levels in rat serum and RAEC supernatant samples were measured using ET-1 ELISA kits (CUSBIO, China) with a biotinylated antibody, HRP-avidin, and tetramethylbenzidine according to the manufacturer’s instructions. The optical density of each sample was measured at 450 nm using a BioTek Synergy H4 microplate reader.

### PKC activity assay

Vascular endothelial samples or RAECs were homogenized with lysis buffer and incubated on ice for 10 min prior to centrifugation at 12,000 *g* for 5 min at 4°C. PKC activity was measured in the resultant supernatants using a specific PKC assay kit (Abcam, ab139437) according to the manufacturer’s instructions. The kinase dilution buffer was incubated with the samples for 90 min at 30°C in the presence of ATP. A phosphorylation-specific antibody, HRP-conjugated secondary antibody, and tetramethylbenzidine (TMB) solution were added successively to the wells, and the optical density of every well was measured at 450 nm using a BioTek Synergy H4 microplate reader. The results were expressed as a percentage of the absorbance values of the normal or control group wells.

### Gene expression analysis

The entire thoracic aorta was dissected, placed on ice, and cut using scissors and the vascular endothelium was obtained by gentle scraping using a cell scraper. RNA was isolated from the vascular endothelium or RAECs using Trizol (Invitrogen) according to the manufacturer’s instructions. cDNA was synthesized using the miScript reverse transcription kit (QIAGEN). Quantitative real-time polymerase chain reaction (qPCR) was conducted using specific primers for miR-199a and miR-138, the miScript SYBR Green PCR kit (QIAGEN), and an ABI 7000 real-time PCR system. The small nuclear RNA U6 was used as an internal control, and the relative expression levels of miR-199a and miR-138 were calculated using the 2^-ΔΔCT^ method [43].

### Western blot analysis

Rats and RAECs were treated in accordance with the respective procedures described in the above relevant subsections. Vascular endothelial cell or RAEC samples were homogenized with lysis buffer and centrifuged at 12,000 *g* for 15 min. Equal amounts of total proteins (40 μg) were separated using sodium dodecyl sulfate-polyacrylamide gel (15%) electrophoresis and then transferred to a polyvinylidene difluoride (PVDF) membrane (IPVH00010, Millipore, Bedford, MA, USA). After blocking with 1% bovine serum albumin for 120 min at 20 ± 2°C, the membranes were incubated with one of the following primary antibodies: anti-HIF-1α (1:1000, ab216842), anti-caspase 3 (1:500, ab32042), anti-eNOS (1:500, ab95254), anti-p-eNOS^Ser1177^ (1:500, ab51038, all Abcam), anti-Bcl-2 (1:1000, SAB4500003), anti-Bax (1:1000, SAB4502546), anti-iNOS (1:1000, SAB4502011), anti-p-iNOS^Tyr151^ (1:1000, SAB4301563), and anti-S100A1 (1:250, SAB4502708, all Sigma-Aldrich) overnight at 4°C prior to washing thrice (15-min each) with Tris-buffered saline containing Tween 20 (TBST).

The blots were then incubated with the corresponding HRP-conjugated secondary antibody for 50–60 min at 20 ± 2°C, followed by three 10-min washes with TBST. Protein bands were visualized using an enhanced chemiluminescence kit (Amersham Corp., Arlington Heights, CA, USA). The relative densities of the protein bands were determined using an image acquisition and analysis system (Leica, Germany). β-actin was used to normalize protein loading. iNOS or eNOS phosphorylation was calculated as the ratio of normalized arbitrary units (a.u.) of p-iNOS or p-eNOS to total iNOS or eNOS, respectively.

### Statistical analysis

Data were presented as the mean ± standard deviation, and statistical comparisons between groups were performed by using a one-way analysis of variance followed by the Student’s *t*-test, using the statistical package for the social sciences (SPSS) v.16.0 software (SPSS, Inc., San Rafael, CA, USA). A *P* < 0.05 was considered statistically significant.

## SUPPLEMENTARY MATERIALS FIGURES



## Abbreviations

Bax: B-cell lymphoma-2-associated X protein
Bcl-2: B-cell lymphoma-2
CBF: cerebral blood flow
EC: endothelial cell
ELISA: enzyme-linked immunosorbent assay
ET-1: endothelin-1
HIF-1α: hypoxia inducible factor-1α
MTT: 3-(4,5-dimethyithiazol-2-yl)-2,5-diphenyl-tetrazolium bromide
NO: nitric oxide
NOS: nitric oxide synthase
OGD: oxygen glucose deprivation
ONOO^-^: peroxynitrite
PKC: protein kinase C
ROS: reactive oxygen species
SYB: safflor yellow B
TUNEL: terminal deoxyribonucleotidyl transferase-mediated dUTP-digoxigenin nick-end labeling

## References

[1] Yetik-Anacak G, Catravas JD (2006). Nitric oxide and the endothelium:history and impact on cardiovascular disease. Vascul Pharmacol.

[2] Wink DA, Hanbauer I, Krishna MC, DeGraff W, Gamson J, Mitchell JB (1993). Nitric oxide protects against cellular damage and cytotoxicity from reactive oxygen species. Proc Natl Acad Sci USA.

[3] Kim YM, Talanian RV, Billiar TR (1997). Nitric oxide inhibits apoptosis by preventing increases in caspase-3-like activity via two distinct mechanisms. J Biol Chem.

[4] Huang PL, Huang Z, Mashimo H, Bloch KD, Moskowitz MA, Bevan JA, Fishman MC (1995). Hypertension in mice lacking the gene for endothelial nitric oxide synthase. Nature.

[5] Shesely EG, Maeda N, Kim HS, Desai KM, Krege JH, Laubach VE, Sherman PA, Sessa WC, Smithies O (1996). Elevated blood pressures in mice lacking endothelial nitric oxide synthase. Proc Natl Acad Sci USA.

[6] Wilcox JN, Subramanian RR, Sundell CL, Tracey WR, Pollock JS, Harrison DG, Marsden PA (1997). Expression of multiple isoforms of nitric oxide synthase in normal and atherosclerotic vessels. Arterioscler Thromb Vasc Biol.

[7] Faller DV (1999). Endothelial cell responses to hypoxic stress. Clin Exp Pharmacol Physiol.

[8] Kisanuki YY, Emoto N, Ohuchi T, Widyantoro B, Yagi K, Nakayama K, Kedzierski RM, Hammer RE, Yanagisawa H, Williams SC, Richardson JA, Suzuki T, Yanagisawa M (2010). Low blood pressure in endothelial cell-specific endothelin 1 knockout mice. Hypertension.

[9] Armstead WM, Riley J, Cines DB, Higazi AA (2014). PAI-1-derived peptide EEIIMD prevents hypoxia/ischemia-induced aggravation of endothelin- and thromboxane-induced cerebrovasoconstriction. Neurocrit Care.

[10] Johansson SE, Larsen SS, Povlsen GK, Edvinsson L (2014). Early MEK1/2 inhibition after global cerebral ischemia in rats reduces brain damage and improves outcome by preventing delayed vasoconstrictor receptor upregulation. PloS one.

[11] Hu J, Discher DJ, Bishopric NH, Webster KA (1998). Hypoxia regulates expression of the endothelin-1 gene through a proximal hypoxia-inducible factor-1 binding site on the antisense strand. Biochem Biophy Res Commun.

[12] Yamashita K, Discher DJ, Hu J, Bishopric NH, Webster KA (2001). Molecular Regulation of the Endothelin-1 Gene by Hypoxia. Contributions of hypoxia-inducible factor-1, activator protein-1, GATA-2, AND p300/CBP. J Biol Chem.

[13] Mateo J, Garcia-Lecea M, Cadenas S, Hernandez C, Moncada S (2003). Regulation of hypoxia inducible factor-1alpha by nitric oxide through mitochondria-dependent and -independent pathways. Biochem J.

[14] Fish JE, Matouk CC, Yeboah E, Bevan SC, Khan M, Patil K, Ohh M, Marsden PA (2007). Hypoxia inducible expression of a natural cisantisense transcript inhibits endothelial nitric-oxide synthase. J Biol Chem.

[15] Bartel DP (2004). MicroRNAs: genomics, biogenesis, mechanism, and function. Cell.

[16] Bartel DP (2009). MicroRNAs: target recognition and regulatory functions. Cell.

[17] Carthew RW, Sontheimer EJ (2009). Origins and mechanisms of miRNAs and siRNAs. Cell.

[18] Most P, Lerchenmüller C, Rengo G, Mahlmann A, Ritterhoff J, Rohde D, Goodman C, Busch CJ, Laube F, Heissenberg J, Pleger ST, Weiss N, Katus HA (2013). S100A1 Deficiency Impairs Postischemic Angiogenesis Via Compromised Proangiogenic Endothelial Cell Function and Nitric Oxide Synthase Regulation. Circ Res.

[19] Rane S, He M, Sayed D, Vashistha H, Malhotra A, Sadoshima J, Vatner DE, Vatner SF, Abdellatif M (2009). Downregulation of miR-199a derepresses hypoxia-inducible factor-1alpha and Sirtuin 1 and recapitulates hypoxia preconditioning in cardiac myocytes. Circ Res.

[20] Yin HL, Luo CW, Dai ZK, Shaw KP, Chai CY, Wu CC (2016). Hypoxia-inducible factor-1α, vascular endothelial growth factor, inducible nitric oxide synthase, and endothelin-1 expression correlates with angiogenesis in congenital heart disease. Kaohsiung J Med Sci.

[21] Cao J, Chen Z, Zhu Y, Li Y, Guo C, Gao K, Chen L, Shi X, Zhang X, Yang Z, Wen A (2014). Huangqi Honghua combination and its main components ameliorate cerebral infarction with Qi deficiency and blood stasis syndrome by antioxidant action in rats. J Ethnopharmacol.

[22] Wang C, Zhang D, Li G, Liu J, Tian J, Fu F, Liu K (2007). Neuroprotective effects of safflor yellow B on brain ischemic injury. Exp Brain Res.

[23] Wang C, He YH, Yang M, Sun HL, Zhang SP, Wang CH (2013). Safflor yellow B suppresses angiotensin II-mediated human umbilical vein cell injury via regulation of Bcl-2/p22phox expression. Toxicol Appl Pharmacol.

[24] Patel N, Gonsalves CS, Malik P, Kalra VK (2008). Placenta growth factor augments endothelin-1 and endothelin-B receptor expression via hypoxia-inducible factor-1 alpha. Blood.

[25] Godinez-Rubi M, Rojas-Mayorquin AE, Ortuno-Sahagun D (2013). Nitric oxide donors as neuroprotective agents after an ischemic stroke-related inflammatory reaction. Oxid Med Cell Longev.

[26] Bogdan C (2001). Nitric oxide and the immune response. Nat Immunol.

[27] Fostermann U, Sessa WC (2012). Nitric oxide synthases: regulation and function. Eur Heart J.

[28] Kulshreshtha R, Ferracin M, Wojcik SE, Garzon R, Alder H, Agosto-Perez FJ, Davuluri R, Liu CG, Croce CM, Negrini M, Calin GA, Ivan M (2007). A microRNA signature of hypoxia. Mol Cell Biol.

[29] Wong DL, Tai TC, Wong-Faull DC, Claycomb R, Siddall BJ, Bell RA, Kvetnansky R (2010). Stress and adrenergic function: HIF1α, a potential regulatory switch. Cell Mol Neurobiol.

[30] Pagé EL, Robitaille GA, Pouysségur J, Richard DE (2002). Induction of hypoxia-inducible factor-1alpha by transcriptional and translational mechanisms. J Biol Chem.

[31] Rouet-Benzineb P, Eddahibi S, Raffestin B, Laplace M, Depond S, Adnot S, Crozatier B (1999). Induction of cardiac nitric oxide synthase 2 in rats exposed to chronic hypoxia. J Mol Cell Cardiol.

[32] Maeno Y, Li Q, Park K, Rask-Madsen C, Gao B, Matsumoto M, Liu Y, Wu IH, White MF, Feener EP, King GL (2012). Inhibition of insulin signaling in endothelial cells by protein kinase C induced phosphorylation of p85 subunit of phosphatidylinositol 3-kinase (PI3K). J Biol Chem.

[33] McQuillan LP, Leung GK, Marsden PA, Kostyk SK, Kourembanas S (1994). Hypoxia inhibits expression of eNOS via transcriptional and posttranscriptional mechanisms. Am J Physiol.

[34] Agani FH, Puchowicz M, Chavez JC, Pichiule P, LaManna J (2002). Role of nitric oxide in the regulation of HIF-1alpha expression during hypoxia. Am J Physiol Cell Physiol.

[35] Saito Y, Nishio K, Ogawa Y, Kinumi T, Yoshida Y, Masuo Y, Niki E (2007). Molecular mechanisms of 6-hydroxydopamine-induced cytotoxicity in PC12 cells: involvement of hydrogen peroxide-dependent and -independent action. Free Radic Biol Med.

[36] Nanetti L, Taffi R, Vignini A, Moroni C, Raffaelli F, Bacchetti T, Silvestrini M, Provinciali L, Mazzanti L (2007). Reactive oxygen species plasmatic levels in ischemic stroke. Mol Cell Biochem.

[37] Kedzierski RM, Yanagisawa M (2001). Endothelin system: the double-edged sword in health and disease. Annu Rev Pharmacol.

[38] Staszel T, Zapała B, Polus A, Sadakierska-Chudy A, Kieć-Wilk B, Stępień E, Wybrańska I, Chojnacka M, Dembińska-Kieć A (2011). Role of microRNAs in endothelial cell pathophysiology. Pol Arch Med Wewn.

[39] Seppey D, Sauser R, Koenigsberger M, Beny JL, Meister JJ (2008). Does the endothelium abolish or promote arterial vasomotion in rat mesenteric arteries? Explanations for the seemingly contradictory effects. J Vasc Res.

[40] McGuire PG, Orkin RW (1987). Isolation of rat aortic endothelial cells by primary explant techniques and their phenotypic modulation by defined substrata. Lab Invest.

[41] Wang XH, Chen SF, Jin HM, Hu RM (2009). Differential analyses of angiogenesis and expression of growth factors in micro- and macrovascular endothelial cells of type 2 diabetic rats. Life Sci.

[42] Pitas RE, Boyles J, Mahiley RW, Bissell DM (1985). Uptake of chemically modified low density lipoproteins *in vivo* is mediated by specific endothelial cells. J Cell Biol.

[43] Cheng HS, Sivachandran N, Lau A, Boudreau E, Zhao JL, Baltimore D, Delgado-Olguin P, Cybulsky MI, Fish JE (2013). MicroRNA-146 represses endothelial activation by inhibiting pro-inflammatory pathways. EMBO Mol Med.

