# Supplementary material for: Safflor yellow B reduces hypoxia-mediated vasoconstriction by regulating endothelial micro ribonucleic acid/nitric oxide synthase signaling
Source: Oncotarget. 2017 Aug 10;8(55):93551–66. doi: 10.18632/oncotarget.20133 (PMC5706818; doi:10.18632/oncotarget.20133)
Supplement: Supplementary file 1 [file oncotarget-08-93551-s001.pdf]

Safflor yellow B reduces hypoxia-mediated vasoconstriction by regulating endothelial micro ribonucleic acid/nitric oxide synthase signaling

SUPPLEMENTARY MATERIALS

A

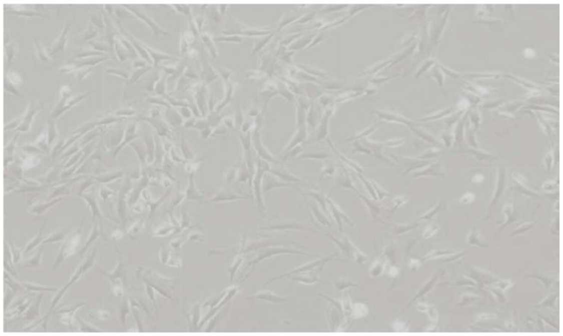

B

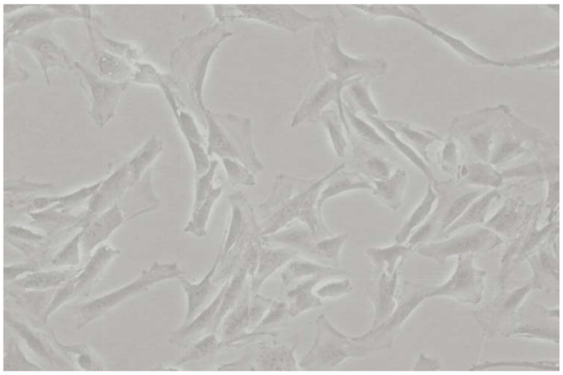

C

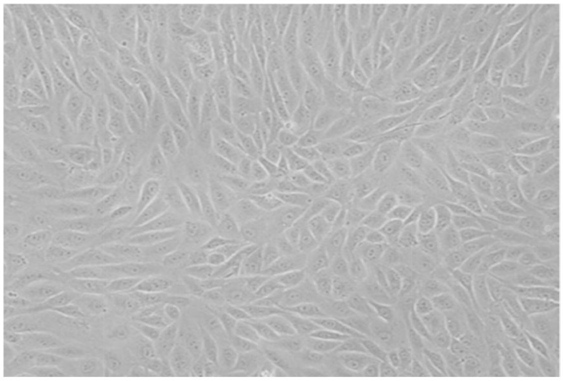

Supplementary Figure 1: Cell morphology: RAECs exhibited a short fusiform or polygonal shape. However, when the cells converged into monolayers, they appeared to have a typical pebble shape.(**A**) 40×; (**B**) 100×; (**C**)100×) Endothelial cell identification: RAECs were investigated by using acetylated low-density lipoprotein(LDL)labeled with 1,1'-dioctadecyl-3,3,3',3'-tetramethyl-indocarbocyanine perchlorate (DiI-Ac-LDL, Molecular probe, Eugene,OR, USA) staining, as described previously [1-5].The procedure as follow: 1. DiI-Ac-LDL method. After DiI-Ac-LDL was engulfed by endothelial cells (ECs), its lipoproteins are degraded by lysosomal enzymes and DiI (fluorescent probe)is accumulated in ECs. We observe the fluorescence intensity of the cells to determine whether the cells belong to ECs by fluorescence microscopy. The procedure as follow: (1) DiI-ac LDL was added directly to the culture medium at a final concentration of 20 ug /ml; (2) Add it to RAECs, and Cells were incubated at 37°C for 4 hours; (3) With a pipette to remove medium; (4) Wash the cells three times with PBS; (5) Fluorescence intensity of cells was observed by fluorescence microscopy at Ex/Em = 549 nm/565 nm.

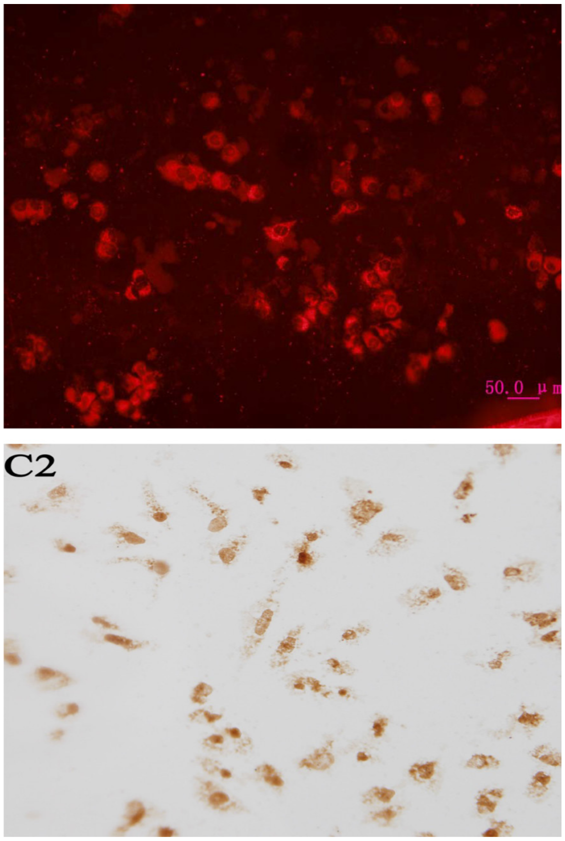

Supplementary Figure 2: Cell morphology: vWF (VIII factor) factor is a characteristic protein of ECs. We measured the expression of vWF (VIII factor) factor by using the SABC method and the result showed that the cultured cells were ECs [6, 2–5].

REFERENCES

1. Pitas RE, Boyles J, Mahiley RW, Bissell DM. Uptake of chemically modified low density lipoproteins in vivo is mediated by specific endothelial cells. J Cell Biol. 1985; 100:103–117.
2. Lamszus K, Schmidt NO, Ergün S, Westphal M. Isolation and culture of human neuromicrovascular endothelial cells for the study of angiogenesis in vitro. J Neurosci Res. 1999; 55:370–381.
3. Miebach S, Grau S, Hummel V, Rieckmann P, Tonn JC, Goldbrunner RH. Isolation and culture of microvascular endothelial cells from gliomas of different WHO grades. J Neurooncol. 2006; 76:39–48.
4. Benten D, Follenzi A, Bhargava KK, Kumaran V, Palestro CJ, Gupta S. Hepatic targeting of transplanted liver sinusoidal endothelial cells in intact mice. Hepatology. 2005; 42:140–148.
5. Li Z, Wu JC, Sheikh AY, Kraft D, Cao F, Xie X, Patel M, Gambhir SS, Robbins RC, Cooke JP, Wu JC. Differentiation, survival, and function of embryonic stem cell derived endothelial cells for ischemic heart disease. Circulation. 2007; 116:46–54.
6. Pan J, Dinh TT, Rajaraman A, Lee M, Scholz A, Czupalla CJ, Kiefel H, Zhu L, Xia L, Morser J, Jiang H, Santambrogio L, Butcher EC. Patterns of expression of factor VIII and von Willebrand factor by endothelial cell subsets *in vivo*. Blood. 2016; 128:104–109.
